# Paraneoplastic dermatomyositis appearing after nivolumab therapy for gastric cancer: a case report

**DOI:** 10.1186/s13256-019-2105-9

**Published:** 2019-06-02

**Authors:** Chikako Shibata, Jun Kato, Nobuo Toda, Makoto Imai, Yukiyo Fukumura, Junya Arai, Ken Kurokawa, Mayuko Kondo, Kaoru Takagi, Kentaro Kojima, Takamasa Ohki, Michiharu Seki, Masanobu Yoshida, Akitake Suzuki, Kazumi Tagawa

**Affiliations:** 1 Department of Gastroenterology, Mitsui Memorial Hospital, 1 Kandaizumicho Chiyoda-ku, Tokyo, 101-8643 Japan; 2 Department of Rheumatology, Mitsui Memorial Hospital, 1 Kandaizumi cho Chiyoda-ku, Tokyo, 101-8643 Japan

**Keywords:** Paraneoplastic dermatomyositis, Nivolumab, IrAE, Anti-TIF1-γ antibody

## Abstract

**Background:**

While dermatomyositis is often associated with malignancy, several autoimmune diseases like myositis can be caused by immune checkpoint inhibitors. Differentially diagnosing malignancy-associated dermatomyositis or myositis caused by immune checkpoint inhibitors is sometimes difficult, particularly when a patient with malignancy shows the symptoms of myositis after checkpoint inhibitor administration. We experienced such a case in which we had difficulties in diagnosing paraneoplastic dermatomyositis or drug-associated myositis. In this case, all of our team initially assumed that the diagnosis was myositis caused by immune checkpoint inhibitors. However, it turned out finally that the diagnosis was paraneoplastic dermatomyositis. Because the diagnosis was unexpected, we report here.

**Case presentation:**

We report the case of a 71-year-old Japanese man who developed clinical symptoms of myositis, such as muscle aches and weakness, after initiation of nivolumab therapy for his gastric cancer. He was initially diagnosed with nivolumab-induced myositis, because the myositis symptoms appeared after nivolumab administration, and nivolumab is known to trigger various drug-associated autoimmune diseases. However, according to his characteristic skin lesions, the type of muscle weakness, his serum marker profiles, electromyography of his deltoid muscle, and magnetic resonance imaging, he was finally diagnosed as having paraneoplastic dermatomyositis. Accordingly, treatment with intravenously administered corticosteroid pulse treatment, immunoglobulin injection, and tacrolimus was applied; his symptoms subsequently improved. However, to our regret, at day 142 after administration, he died due to rapid worsening of his gastric cancer.

**Conclusion:**

Differentially diagnosing paraneoplastic dermatomyositis or drug-associated myositis caused by immune checkpoint inhibitors is difficult in some cases. The differential diagnosis is crucial because it influences the decision regarding the appropriateness of the use of immunosuppressive treatment against the autoimmune diseases as well as the decision regarding the appropriateness of the continuous use of immune checkpoint inhibitors against the primary cancers. Because subclinical autoimmune disease may become overt after administering immune checkpoint inhibitors, non-apparent autoimmune diseases, which have already existed, should also be considered to avoid the delay of appropriate treatment, when symptoms of autoimmune diseases are recognized.

## Background

While dermatomyositis has a high risk of malignancy, myositis can be induced by malignancy as well as by drugs [1]. Nivolumab is an immune checkpoint inhibitor effective against various types of cancer and one of the drugs that cause myositis as well as other adverse events, known as immunotherapy-related adverse events (irAEs) [2].

The programmed cell death protein 1 (PD-1)/programmed death-ligand (PD-L) system plays an important role in tumor immunity. Expression of PD-L1, a PD-L expressed on the cell surface of tumor cells, weakens the function of cytotoxic T cells, which attack tumor cells. Nivolumab, a PD-1 blocker, inhibits the PD-1/PD-L pathway, activates cytotoxic T cells, and suppresses the proliferation of the cancer cells. Simultaneously, nivolumab activates T cells and attacks normal cells, which causes various autoimmune disorders in some cases. In fact, mice genetically lacking PD-1 develop spontaneous autoimmune diseases [3, 4].

Here we report a case with advanced gastric cancer who developed symptoms of myositis after initiation of nivolumab as the third-line chemotherapy, which was difficult to diagnose as paraneoplastic dermatomyositis or drug-associated myositis. The final diagnosis was paraneoplastic dermatomyositis, which was unexpected. We should be aware that there are such cases in which pre-existing subclinical paraneoplastic symptoms become apparent after immunotherapy when using immune checkpoints inhibitors.

Because nivolumab is being applied to more and more cases of cancer, we may confront similar difficulties in the future. Here, we report how to reach the diagnosis, which will help physicians diagnose cases with similar symptoms after administering nivolumab.

## Case presentation

**Fig. 1:**
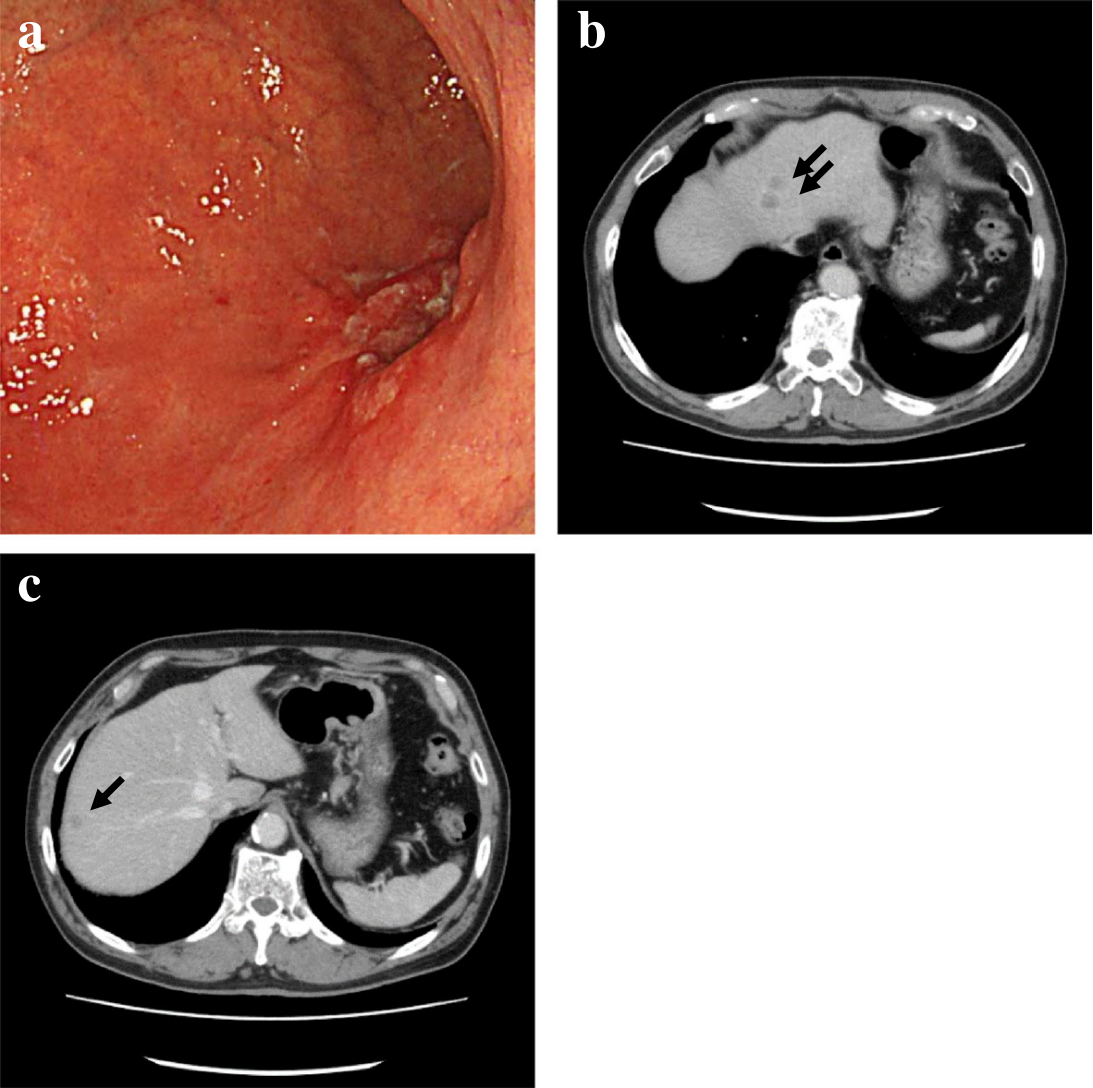
Gastrointestinal endoscopy and computed tomography findings at the time of diagnosis of gastric cancer. **a** Endoscopic view of advanced gastric cancer on the posterior wall of the lower part of his stomach. **b**, **c** Liver metastases (*arrows*) detected by computed tomography

A 71-year-old Japanese man with no notable medical history, including autoimmune disease, was diagnosed as having gastric cancer and liver metastases in April 2017 (Fig. 1a–c): T2N0M1 cStage IV; Union for International Cancer Control (UICC) 7th edition. His maternal grandfather and his brother had gastric cancer. He drank 360 ml of rice wine every day and smoked approximately 50 cigarettes/day until 24 years ago.

Since May 2017, he received first-line chemotherapy comprising four cycles of cisplatin and tegafur, gimeracil, and oteracil potassium, followed by four cycles of second-line therapy with paclitaxel and ramucirumab. Although his liver metastases shrank, in December 2017 (that is, at 7 months after the initiation of chemotherapy) the primary tumor and metastases were found to have progressed.

He had no history of apparent autoimmune disease; his serum autoimmune disease-related markers were negative. During the second-line chemotherapy, his anterior chest and dorsal surfaces of his fingers became reddened, which was considered to be caused by ramucirumab. He had no difficulties in drinking or swallowing solid matter, and showed no obvious neurological dysfunction. Because it seemed he had no evidence that he had autoimmune disease, including dermatomyositis, we decided to initiate nivolumab as the third-line therapy.

Nivolumab (3 mg/kg) was administered in January 2018. A few hours after administration, he developed fever of 38 °C, which gradually dropped down to low-grade fever. Two weeks after administration he visited our hospital for the second treatment with nivolumab, he said he had been suffering from general fatigue, difficulty in swallowing, muscle aches, low-grade fever, face edema, and erythema of the nose, anterior chest, and dorsal surfaces of his fingers. He could easily drink water, but had difficulties in swallowing solid materials. He showed no obvious neurological dysfunction and had no trouble in walking by himself.

Blood tests showed an increase in the levels of creatine phosphokinase (CK; 300 U/L), aspartate aminotransferase (AST; 37 U/L), myoglobin (354 ng/mL), and C-reactive protein (CRP; 3.32 mg/dL). Before nivolumab administration, his CRP level had been slightly high (around 1.0 mg/dL), and his CK and AST levels had been within normal range. Because drug-associated myositis was suspected, he was immediately admitted to our hospital.

After admission, the difficulty in swallowing worsened at the end of January (approximately 20 days after nivolumab administration) and his CK and AST levels gradually increased to > 1000 and > 100, respectively, at 22 days after administration. Because of the worsening of his general condition, nivolumab treatment was suspended. Instead of nivolumab, treatment with prednisolone (0.5 mg/kg, 30 mg/body per day) was initiated at 22 days after nivolumab administration, as frequently applied for drug-induced dermatomyositis [5].

However, the results of his blood test did not improve, and his general condition worsened. In late February (approximately 40 days after nivolumab administration), he became unable to stand up or eat. The skin lesion had spread over his ears, elbows, and knees, as well as his face, right shoulder, anterior chest, left hip, and fingers. A neurological examination indicated dysphagia, muscle weakness (mainly proximal), and depression of the tendon reflexes of his extremities. Because we considered that the steroid treatment (30 mg/body per day) was ineffective and that long-term high-dose prednisolone was harmful, we reduced the dose of prednisolone to 20 mg/body per day 41 days after starting nivolumab.

**Fig. 2:**
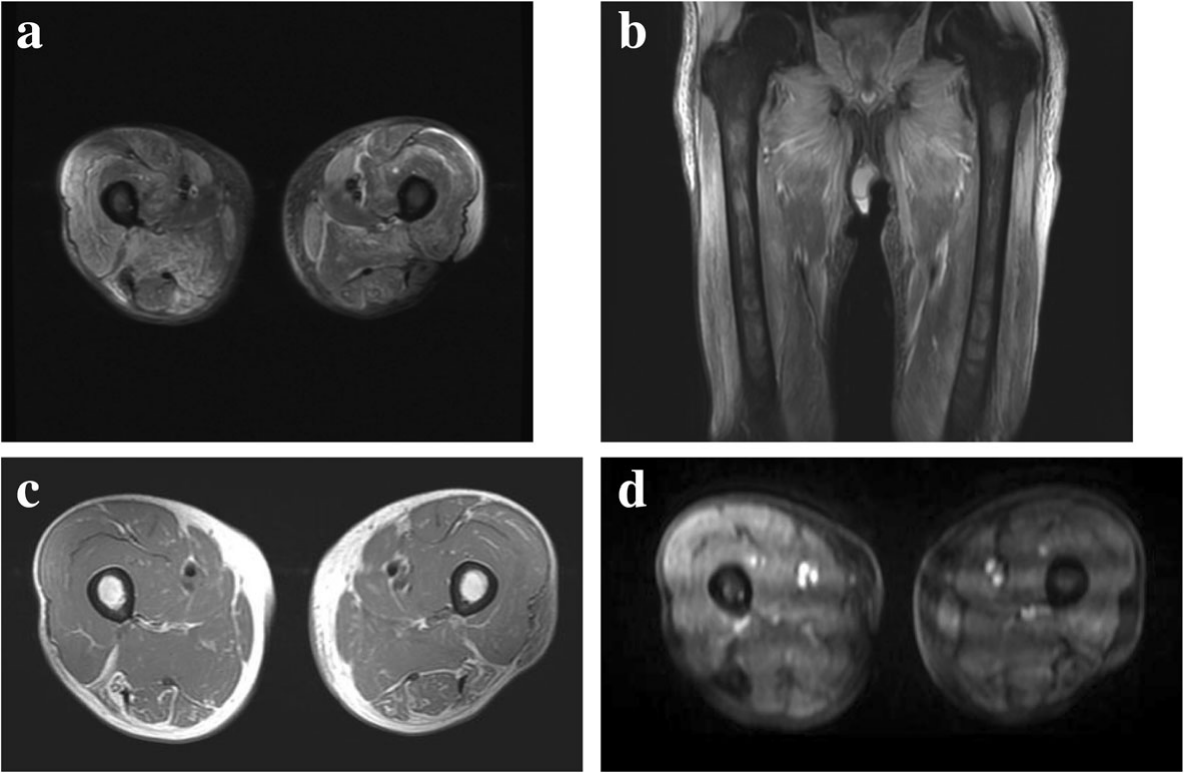
Myositis detected by magnetic resonance imaging. **a**, **b** Magnetic resonance imaging of the bilateral femoral shows a heterogeneous high signal intensity in the bilateral femoris muscles in short-T1 inversion recovery images. **c** T1 image before enhancement. **d** A heterogeneous contrast enhancement in the bilateral femoris muscles in gadolinium-enhanced T1 images

More examinations were needed to identify his disease condition correctly. Electromyography of his deltoid muscle and biceps brachii revealed a low motor unit potential (MUP), and magnetic resonance imaging (MRI) showed a heterogeneous high signal intensity in the bilateral femoris muscles in short-T1 inversion recovery (STIR) images, and a heterogeneous contrast enhancement in the bilateral femoris muscles (mainly proximal) in gadolinium-enhanced T1 images; these findings are suggestive of myositis (Fig. 2). The condition of our patient (skin lesion, muscle weakness, elevation of CK level and CRP level, and electromyography findings) fulfilled the diagnostic criteria for dermatomyositis. A muscular biopsy was not performed because it was too invasive to our patient. Although he was negative for anti-Jo-1, −Scl-70, −RNP, −Sm, −Mi-2, −MDA5, and −ARS antibodies, his antinuclear antibody index was 80 and his anti-transcriptional intermediary factor 1-γ (TIF1-γ) antibody index was 111 (normal range < 32). Because anti-TIF1-γ is a marker for paraneoplastic dermatomyositis, our patient was suspected to have paraneoplastic dermatomyositis rather than nivolumab-induced myositis. Also, his reddened anterior chest and dorsal surfaces of his fingers before the initiation of nivolumab, which we had considered a ramucirumab-induced eruption, were actually the V-neck sign and Gottron’s papule on his fingers. The erythema of his fingers was hyperkeratotic, erythematous, flat papules with central atrophy, and on the dorsum of his metacarpophalangeal and interphalangeal joints, all of which were compatible with the symptoms of dermatomyositis [6]. Therefore, his diagnosis was changed from nivolumab-induced myositis to paraneoplastic dermatomyositis.

**Fig. 3:**
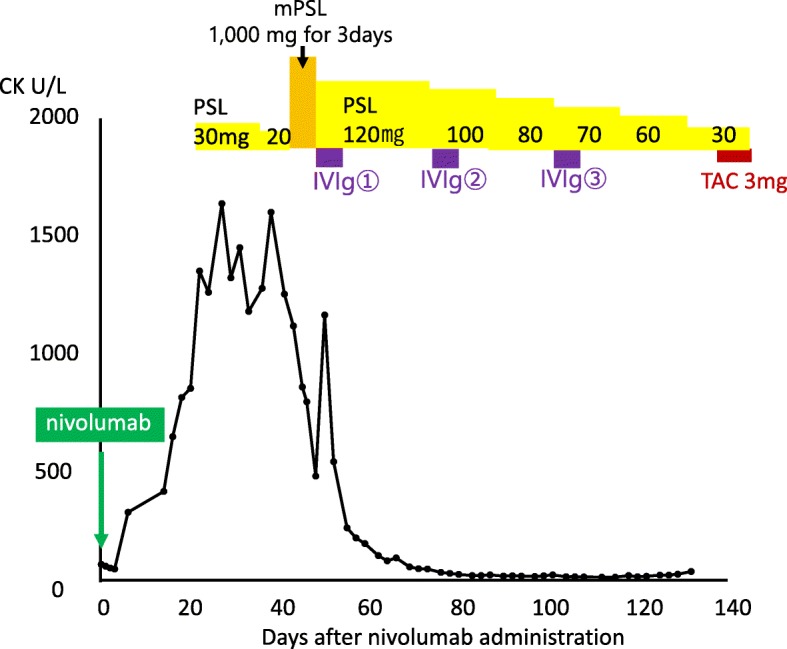
Clinical course of the patient after nivolumab therapy. The patient’s creatine phosphokinase level increased to > 1000 at 22 days after nivolumab administration, when treatment with prednisolone (30 mg/body per day) was initiated. Because his creatine phosphokinase level did not improve, steroid pulse therapy and intravenously administered immunoglobulin were initiated. His creatine phosphokinase level subsequently decreased markedly and his symptoms improved. After tapering prednisolone to 30 mg/day, tacrolimus was applied to maintain the improved status. *CK* creatine phosphokinase, *IVIg* intravenous immunoglobulin, *mPSL* methylprednisolone, *PSL* prednisolone, *TAC* tacrolimus

Because we assessed that the applied treatment of prednisolone (30 mg/body per day, tapering to 20 mg/body per day) was not effective, according to the standard treatment for resistant or severe paraneoplastic dermatomyositis [7, 8], steroid pulse treatment (methylprednisolone 1000 mg/day for 3 days, followed by tapering prednisolone from 120 mg/day to 30 mg/day at 129 after nivolumab administration) and intravenously administered immunoglobulin 30 g/day (400 mg/kg per day) for 5 days once a month three times beginning on day 45 after nivolumab administration were initiated. Our patient’s CK level rapidly decreased in response to the treatment (Fig. 3), and his physical activity significantly improved. At 2 weeks after steroid pulse treatment initiation, he could walk by himself, and at 6 weeks he could walk smoothly and stand up with little help. While chemotherapy for his gastric cancer was not applied during the treatment of paraneoplastic dermatomyositis, his general condition improved, and his rehabilitation continued. Despite the improvement of his general condition, the dysphasia was not improved and he could not eat or drink. A nasogastric tube was inserted at day 128 for his nutrition, and tacrolimus, an immunosuppressive agent that is frequently used for resistant or severe dermatomyositis, was administered through it from day 129 after nivolumab administration. We tried to make it possible for him to be discharged from our hospital.

However, his general condition drastically worsened at day 130 after first nivolumab administration. To our regret, at day 142 after administration, he died due to rapid worsening of his gastric cancer. After his death, an autopsy was performed. According to the interim report, the cancer had progressed more than expected. The tumor spread throughout his whole body, and metastasized to his liver, lung, and abdominal cavity. Although muscles were normal in gross appearance, a pathological examination is still under investigation.

## Discussion

We report a case with paraneoplastic dermatomyositis that became apparent after the initiation of nivolumab. We initially suspected nivolumab-induced dermatomyositis. However, because our patient’s TIF1-γ antibody level was high, and he had Gottron’s papule and other skin lesions before nivolumab treatment, we diagnosed paraneoplastic dermatomyositis in the end.

Dermatomyositis carries a risk of malignancy; around 9–30% of patients with dermatomyositis have malignancies, 2.4–5-fold higher than the rate in the normal population [9–11]. Patients with dermatomyositis who are anti-TIF1-γ antibody-positive are at particularly high risk for malignancy [12, 13]. Although it is hard to deny that he had nivolumab-associated dermatomyositis with the high anti-TIF1-γ antibody titer, his course, the presence of skin lesions before nivolumab therapy, strongly suggested that the diagnosis was paraneoplastic dermatomyositis.

Nivolumab is a human immunoglobulin G4 monoclonal antibody to PD-1, which blocks malignant signaling. Nivolumab is the first immune checkpoint inhibitor effective against advanced gastric or gastroesophageal junction cancer [14]. In fact, nivolumab has recently been approved for use as the third-line chemotherapy in patients in Japan with gastric or gastroesophageal junction cancer.

The known irAEs of nivolumab include pneumonitis, acute respiratory distress, myositis, colitis, hypothyroidism, hypophysitis, and skin rash [2, 15, 16]. Although various autoimmune-related adverse events related to nivolumab have been reported, nivolumab-triggered dermatomyositis, apart from myositis, is rare and there is only one case in the literature, as far as we know. Kudo *et al.* reported the case of a patient who was treated with nivolumab for his advanced lung adenocarcinoma and was diagnosed as having nivolumab-associated dermatomyositis from his symptoms and his examinations [17]. They proposed it is necessary to consider drug-associated dermatomyositis even after nivolumab therapy [17]. In our case, however, our patient was finally diagnosed not as having nivolumab-induced myositis but as having paraneoplastic dermatomyositis because of the time course of redness of skin and the high anti-TIF1-γ antibody titer.

Patients with a pre-existing autoimmune or inflammatory disease are at high risk of irAEs. Although we administered nivolumab after confirming that there was no history of apparent autoimmune disease and his serum autoimmune disease-related markers were negative, subclinical paraneoplastic dermatomyositis became apparent after nivolumab and it turned out that he had paraneoplastic dermatomyositis. Because, as shown in this case, the symptoms usually rapidly improve after appropriate treatment, we must be very cautious about the possibilities of the existence of autoimmune diseases before applying immune checkpoint inhibitors.

## Conclusion

Differentially diagnosing paraneoplastic dermatomyositis or drug-associated myositis caused by immune checkpoint inhibitors is difficult in some cases. We experienced such a difficult case in which pre-existing subclinical paraneoplastic dermatomyositis became apparent after initiation of nivolumab therapy. Existence of non-apparent immune-related diseases should be always considered before and after applying immune checkpoint inhibitors. In addition, appropriate interventions should be applied correctly and immediately upon symptoms becoming apparent. Our report here will help physicians to diagnose cases with similar symptoms correctly after administration of immune checkpoint inhibitors.

## Abbreviations

AST: Aspartate aminotransferase
CK: Creatine phosphokinase
CRP: C-reactive protein
irAEs: Immunotherapy-related adverse events
MRI: Magnetic resonance imaging
MUP: Motor unit potential
PD-1: Programmed cell death protein 1
PD-L: Programmed death-ligand
STIR: Short-T1 inversion recovery
TIF1-γ: Transcriptional intermediary factor 1-γ
UICC: Union for International Cancer Control
